# Dye-Doped Polymeric Microplastics: Light Tools for Bioimaging in Test Organisms

**DOI:** 10.3390/polym15153245

**Published:** 2023-07-30

**Authors:** Federica Bertelà, Chiara Battocchio, Giovanna Iucci, Simona Ceschin, Dario Di Lernia, Flaminia Mariani, Andrea Di Giulio, Maurizio Muzzi, Iole Venditti

**Affiliations:** 1 Department of Sciences, Roma Tre University, Via della Vasca Navale 79, 00146 Rome, Italy; federica.bertela@uniroma3.it (F.B.); chiara.battocchio@uniroma3.it (C.B.); giovanna.iucci@uniroma3.it (G.I.); simona.ceschin@uniroma3.it (S.C.); dar.dilernia@stud.uniroma3.it (D.D.L.); flaminia.mariani@uniroma3.it (F.M.); andrea.digiulio@uniroma3.it (A.D.G.); maurizio.muzzi@uniroma3.it (M.M.); 2 NBFC National Biodiversity Future Center, 90133 Palermo, Italy

**Keywords:** polymeric microparticles, plastic pollution, rhodamine B isothiocyanate, fluorescein isothiocyanate, dye-doped polymers, bioimaging

## Abstract

Ecosystems around the world are experiencing a major environmental impact from microplastic particles (MPs 0.1 µm–1 mm). Water, sediments, and aquatic biota show the widespread presence of this pollutant. However, MPs are rarely used in laboratory studies as they are scarcely available for purchase or expensive, especially if one wishes to trace the particle with a dye or fluorescent. Furthermore, existing preparation techniques have limited application in biological studies. In this work, we propose a new, easy, and cheap way to prepare fluorescent MPs. The protocol is based on the osmosis method in order to obtain spherical polymeric particles of P(S-co-MMA), with 0.7–9 micron diameter, made fluorescent because dye-doped with rhodamine B isothiocyanate (RITC) or fluorescein isothiocyanate (FITC). The dye loading was studied and optimized, and the MPs–dye conjugates were characterized by UV-vis FTIR and XPS spectrometry and scanning electron microscopy (SEM). Furthermore, preliminary tests on aquatic organisms demonstrated the possible use of these fluorescent MPs in bioimaging studies, showing their absorption/adsorption by duckweeds (*Lemna minuta*) and insect larvae (*Cataclysta lemnata*).

## 1. Introduction

In the last decade, nano- and microstructured polymeric materials are finding wide use in different fields, such as optics, sensing, and biomedical applications [1,2,3,4,5,6,7,8].

In many cases, these materials combine typical properties of polymers, such as synthetic versatility, workability, and the ability to form films, with the optical or biological properties of organic/inorganic molecules used as dopants. Furthermore, the possibility of being nano- and microstructured through top-down or bottom-up approaches gives these hybrid systems the well-known advantages related to the high surface/volume ratio and size scale comparable to cells and biomolecules [9,10,11,12,13,14].

The top-down approach is essentially the breaking down of bulk material to obtain nano- and microsized particles. This can be achieved by using advanced techniques such as precision engineering and lithography which have been developed and optimized by industry during recent decades. Precision engineering supports much of the microelectronics industry during the entire production process, and high performance can be achieved using a combination of improvements. These include the use of advanced nano- and microstructures based on diamond or cubic boron nitride and sensors for size control, combined with numerical control and advanced servo-drive technologies [15,16,17]. Lithography involves the patterning of a surface through exposure to light, ions, or electrons, and the deposition of material onto that surface to produce the desired material [18,19,20]. The OBM or dialysis method has been successfully applied in the preparation of polymeric nano- and microparticles. The method is based on the use of dialysis membranes with a suitable molecular weight (MWCO) which act as a physical barrier for the polymer. Generally, the polymer is dissolved in an organic solvent, then placed within the dialysis membrane and dialyzed against a non-solvent. The displacement of the non-solvent within the membrane causes the mixture to become progressively less capable of dissolving the polymer. Furthermore, an increase in interfacial tension causes polymer aggregation and leads to the formation of a colloidal suspension of nanoparticles. The basic prerequisite is the miscibility of solvent and non-solvent. The method is applicable to any polymer, commercial or synthetic, or copolymers, provided that the solvent/non-solvent and the appropriate concentrations are chosen in an adequate manner. This method allows definition and modulation of the shape and dimensions by varying the experimental parameters, such as the solvent/non-solvent pair, MWCO of dialysis, the temperature at which the procedure is performed, the polymer concentration, and the solvent mixing rate [21]. Akagi et al. [22] investigated the influence of solvent by analyzing four important organic solvents, namely dimethyl sulfoxide (DMSO), dimethylformamide (DMF), dimethylacetamide (DMAc), and N-methyl-2-pyrrolidinone (NMPy) capable of dissolving poly(γ-glutamic acid) (PGGA). The particles prepared with DMSO were smaller, with a narrower size distribution than those prepared with NMPy. A similar approach was used by Jeong et al. [23] for preparation of PLGA nanoparticles from DMAc, DMF, DMSO, and acetone as polymer solvents. The sizes of the PNPs prepared from DMAc, DMF, and DMSO were between 200 and 300 nm and not significantly different. On the other hand, acetone produced larger particles with an average size of 642 nm. This change in particle size could be explained by the differences in solvent viscosity, water miscibility, and solubility behavior of the polymer.

The bottom-up approach refers to the buildup of nano- and microstructures from the bottom: atom by atom or molecule by molecule by physical and chemical methods in a nano-, meso-, and microscale range (1–100 nm, 100–1000 nm, and 1–10 µm, respectively) using controlled manipulation of self-assembly of atoms and molecules. Chemical synthesis is a method of producing rough materials that can be used either directly in products in their bulk disordered form, or as building blocks of more advanced ordered materials. Self-assembly is a bottom-up approach in which atoms or molecules organize themselves into ordered nano- and microstructures by chemical–physical interactions. Positional assembly is the only technique in which single atoms, molecules, or clusters can be positioned freely one by one [24,25,26]. In particular, in defining the functional properties of the material, the structure of the dispersed phase and the dispersion of the organic molecules within the polymeric matrix must be taken into account. Equally important are the chemical–physical characteristics of the polymeric matrix; its use not only can stabilize colloidal dispersions by preventing aggregation phenomena but also acts on their processability by improving their technological properties such as solubility and thermal stability. Emulsion techniques, in numerous variants, have been widely used to produce monodispersed polymeric nano- and microparticles which often gave rise to self-assembly. Photonic crystals with applications in optics and sensors are well known, including artificial opals that can be realized in a direct or inverse way [2,3,7]. It is well known that dispersing a liquid in the form of droplets in another liquid with surfactants creates an emulsion. Emulsion droplets can act as containers on the nano- or micro-scale, using their interfaces, stabilized with suitable surfactant, as permeable walls to perform chemical–physical processes, showing great power in creating functional nano- or microparticles. In general, accurate droplet size control with high uniformity in the nano- or micrometer range can provide each droplet with a desirable volume and interface area for encapsulation of substances, and mass and heat transfer, in a highly predictable and efficient way. The controllable manipulation of shape, structure, and composition, especially the control of the separated phase structure of multiple emulsions and the composition in each of their separate liquid phases, offers high flexibility for spatial engineering of different functional components within a drop, allowing versatile design of nano- or microparticles [2,3].

Among others, poly(styrene-co-methyl methacrylate) [P(S-co-MMA)] is a copolymer widely studied in bottom-up and top-down approaches. It has properties which are intermediate between those of polystyrene (PS) and polymethylmethacrylate (PMMA): PS displays low modulus, excellent abrasion resistance, acceptable load-bearing resistance, and high tensile strength, whereas PMMA exhibits good transparency, high modulus and melt viscosity, but low abrasion and resistance to wear. The copolymer combines the properties of the two polymers into a single polymer and it displays synergically enhanced chemical–physical properties. These improved properties enable its use in various fields, such as medicine, smelting, and the automotive industry [27].

In the past five years, studies on the environmental impact of these microplastic materials have been carried out, in which suitably selected and prepared particles were studied to verify their toxicity and ecotoxicity to plants and animals [28,29,30]. In this way it has been possible to reproduce in laboratory tests the impact that nano- and microplastics can have on plants and animals, tracing them through the presence of fluorescent dopants [31,32,33,34]. In recent environmental studies, bioimaging attempts have been made to trace specific pollutants in biological systems (especially metals and plastics) to investigate their potential implications regarding the link between localization and functionality, key studies in ecotoxicology [35,36]. Addressing such important questions certainly requires technological advances in capturing images at the nano-, micro- and macroscales. However, to date, the bottleneck is the offer to the scientific community of easy and cheap technologies.

In this work, using a top-down approach, we prepared polymeric microparticles (MPs) based on P(S-co-MMA), with diameters in the range 0.7–9 µm, doped with two different organic dyes, i.e., rhodamine B isothiocyanate (RITC) and fluorescein isothiocyanate (FITC). The dye-doped MPs, i.e., FITC-MPs and RITC-MPs, were fully characterized by means of UV-vis, FTIR, and XPS spectroscopies and SEM. The aim was to optimize the dye content, maintaining adequate control of shape, dimension, and polydispersity of these MPs in view of their use as bioimaging tools in ecotoxicological studies to evaluate the environmental impact of microplastics on model plants and animals.

## 2. Materials and Methods

### 2.1. Materials

All the solvents (dimethylformamide, acetone) and P(S-co-MMA) (Aldrich 462896, average Mw 100,000–150,000, pellets, styrene 40%) were purchased from Aldrich; all the solvents were technical grade. The dialysis cellulose membrane (width 10 mm, Sigma Aldrich D9277-100FT) was from Aldrich. Rhodamine B isothiocyanate (RITC, 90% pure) and fluorescein isothiocyanate (FITC, 90% pure) were purchased from Aldrich.

### 2.2. Dye-Doped Polymeric Microplastic Particles Preparation

The polymeric beads were obtained starting from pellets of P(S-co-MMA) (Aldrich 462896, average Mw 100,000–150,000, pellets, styrene 40%) following the OBM method reported in a previous work [37]. Specifically, an appropriate amount of P(S-co-MMA) was dissolved in an appropriate volume of solvent, i.e., acetone and dimethylformamide, (DMF) and stirred for 24 h. To obtain the fluorescent MPs, dye was put into the flask with P(S-co-MMA), dissolved by solvent, and stirred for 24 h. After this time, an aliquot of 7 mL of solution was transferred into a dialysis cellulose membrane (width 10 mm, Sigma Aldrich D9277-100FT) and further immersed into 200 mL of distilled water for 5 days at constant temperature (T = 25 °C) (for details see Supporting Information Table S1). The best dye loading was obtained dissolving 3 mg of dye with P(S-co-MMA) in acetone, analogous to our previous studies (details in Supporting Information Table S2), evaluated using calibration curves (see Supporting Information Figure S1a,b), and the loading efficacy derived using:

η (%) = (mg _dye loaded_/mg _dye_) × 100

### 2.3. Dye-Doped Polymeric Microplastic Particles’ Characterizations

Dye-doped MPs were investigated by SEM (Gemini 300, Carl Zeiss AG, Jena, Germany), and their mean diameter was calculated by 100 measurements performed on the same sample using ImageJ software vers. 1.53t (National Institutes of Health, Bethesda, MD, USA) directly on SEM images. The preparation of dye-doped MP samples for SEM observation included samples’ mounting on a stub (using self-adhesive carbon discs) followed by gold sputter-coating (Emitech k550), with subsequent observation by SEM. The particle size distribution is expressed by the ratio Dw/Dn, i.e., polydispersity index (PI). Dw and Dn are the weight and the number average diameter of particles, respectively. FTIR measurements were performed by means of a VECTOR 22 (Bruker) FTIR interferometer operating in the wavenumber range 400–4000 cm^−1^ (resolution 1 cm^−1^) and equipped with a DTGS detector. Samples of dye-doped MPs and of pristine FITC and RITC were prepared as KBr pressed pellets. Thin films of P(S-co-MMA) were prepared by casting from CHCl_3_ solution onto reflective gold surfaces; spectra of P(S-co-MMA) were recorded with a Specac P/N 19650 series monolayer/grazing angle accessory at 70° incidence angle of impinging radiation with respect to the normal sample surface. XPS analysis of FITC-MP and RITC-MP samples was performed with a homemade instrument, consisting of preparation and analysis UHV chambers separated by a gate valve. The analysis chamber was equipped with a 6-degrees-of-freedom manipulator and a 150 mm mean radius hemispherical electron analyzer with a 5-lens output system combined with a 16-channel detector, giving a total instrument resolution of 1.0 eV as measured at the Ag 3d_5/2_ core level. Samples were introduced in the preparation chamber and left for outgassing overnight at a base pressure of about 10^−8^ Torr, before introduction into the analysis chamber. Typical vacuum pressure in the analysis chamber during measurements was in the 10^−8^–10^−9^ Torr range. The X-ray radiation used was a non-monochromatized MgKα (1253.6 eV). Calibration of the energy scale was made referencing the spectra to the C1s core-level signal of aliphatic C atoms, found at 285.0 eV, for all samples. Curve-fitting analysis of the C1s, N1s, O1s, and S2p spectra was performed using Gaussian profiles as fitting functions, after subtraction of a polynomial background. The S2p_3/2_-S2p_1/2_ doublets were fitted using the same full width at half maximum (FWHM) for the two spin-orbit components of the same signal, a spin-orbit splitting of 1.20 eV, and the branching ratios S2p_3/2_/S2p_1/2_ = 2/1. When several different species were identified in a spectrum, the same FWHM value was set for all individual photoemission bands.

### 2.4. Bioimaging in Test Organism

Organisms for bioimaging tests were chosen from those used as model organisms in studies on the trophic transfer of MPs from producers (plants) to primary consumers (herbivores): the aquatic plant *Lemna minuta* and aquatic larvae of the insect *Cataclysta lemnata* [38,39]. These organisms were exposed to tap water contaminated with RITC-MPs (100 mL of suspension of RITC-MPs 100 mg/L), in the same conditions discussed in our previous works (28 days for *L. minuta* and 21 days for *C. lemnata*, grown in the presence of *L. minuta* fronds contaminated with RITC-MPs) [38,39]. After these exposures, specimens of the two organisms were observed under a macroscope (Axiozoom v16, Zeiss) equipped with a HXP 200C metal halide lamp, PlanNeoFluor Z 1x objective, and a color photocamera (Axiocam 503, Zeiss). A HE DsRed 43 filter (excitation: 550/25 nm, emission: 605/70 nm) was used for observing the possible interaction of the fluorescent MPs with the body surfaces of these organisms and, therefore, tracing the presence of the microplastic contaminant.

## 3. Results and Discussion

### 3.1. Dye-Doped MPs: Preparation and SEM Characteriztion

**Scheme 1:**
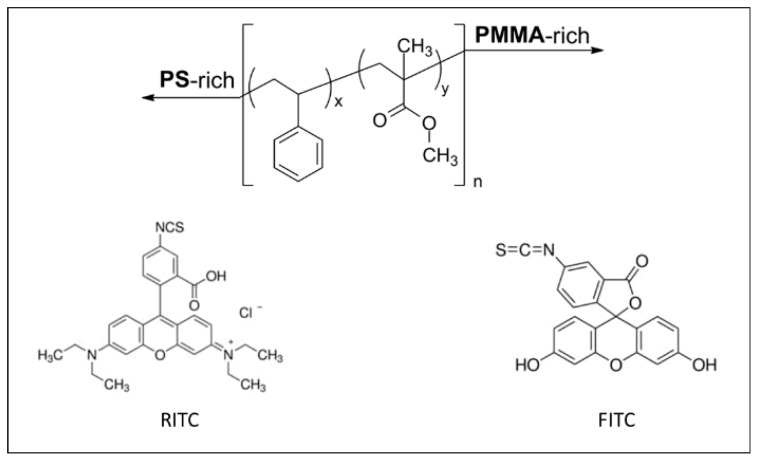
Scheme of chemical structures of P(S-co-MMA), RITC, and FITC.

The MPs were prepared using an osmosis-based method (OBM) that is a simple and versatile procedure for the control of both dimension and morphology of polymeric materials at the micro- and sub-micrometric scale [21,40,41]. The OBM uses a physical barrier, specifically dialysis membranes or common semipermeable membranes, that allows the passive transport of solvents (acetone or DMF in this case) to slow down the mixing of a polymer solution with a non-solvent (water in this case). The shape, in our case spheroidal, and resulting size of the polymeric microparticles produced depends on the chemical–physical conditions that allow minimization of the internal energy. The morphology is conditioned by thermodynamic and kinetic factors, as can be seen from the literature [42]. However, by making the precipitation process take place slowly, the kinetic parameters can be neglected and thermodynamic control can be maintained. The interface free energy is the main thermodynamic parameter and is minimized by the assembly of the polymer chains in a spherical shape. This method has various advantages: low cost, general applicability, mild conditions, obtaining pure products, solvents that are easily recovered by distillation, and almost quantitative yields. Moreover, the method permits microparticle formation simultaneously with the molecule immobilization. In this work, for the first time, fluorescent dyes, i.e., RITC and FITC, were included in MPs (see Scheme 1).

Furthermore, by keeping the temperature constant at 25 °C and ensuring thermodynamic control, a modulation of the dimensions can be obtained by varying the experimental conditions, such as the type of solvent and concentration of the polymeric molecole. The data reported in Table 1 show how, with the same DMF solvent, spheres of smaller dimensions were obtained at lower concentrations of P(S-co-MMA). The same situation was realized using acetone. The lower concentration allowed both to have higher nucleation points, but above all, lower coalescence of the particles in formation. The particle size distribution is expressed by the ratio Dw/Dn, namely, the polydispersity index (PI). Dw and Dn are, respectively, the weight average diameter and the number average diameter of the particles, and were calculated using 50 measurements taken from the SEM images of the same sample.

**Figure 1:**
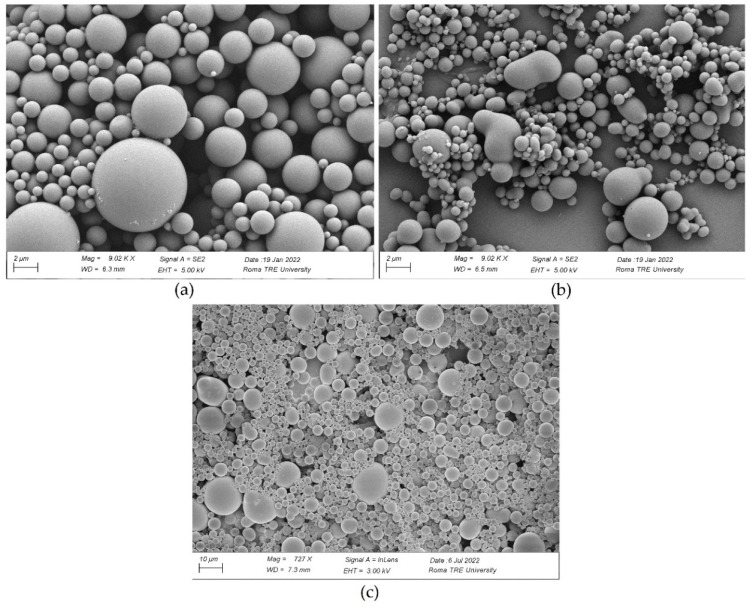
SEM images of P(S-co-MMA) micro and sub-microparticles obtained by OBM, at 25 °C, with 0.3 mg: (**a**) in acetone/H_2_O; (**b**) in DMF/H_2_O; (**c**) in acetone/H_2_O with RITC.

The difference in dielectric constant of the solvent/non-solvent (Δε) also influences the size and polydispersity of the precipitate. In our case, in which the copolymer was made up of apolar styrene and medium polar methyl methacrylate, the morphology was irregular, sometimes like unresolved or fused spheres with the DMF/H_2_O pair having Δε = 42. Instead, for the pair acetone/water with Δε = 58 the morphologies are regular spheres, as shown in Figure 1a,b.

Having studied and verified the appropriate conditions to obtain spherical shapes in an appropriate dimensional range, we moved on to inserting the dye. From among other dyes, RITC and FITC were chosen due to their wide use in the biotechnological field, linked to optical, biomedical, and environmental studies [43,44,45].

The procedure followed to incorporate the dyes into the MPs allowed a good loading efficiency to be obtained, η (%) = 99% and 96% for RITC and FITC, respectively, maintaining suitable control over dimensions and PI, in particular for RITC-doped MPs (see Figure 1c and Table S2 in Supporting Information).

### 3.2. Dye-Doped MPs: Structural Characterizations

**Figure 2:**
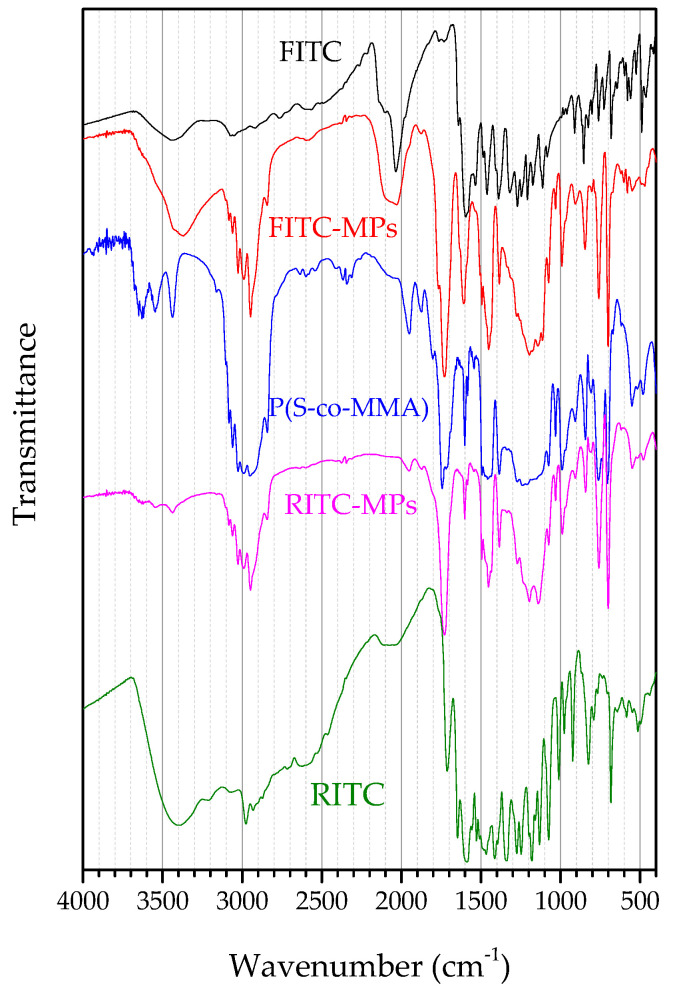
FTIR spectra of dye-doped microparticles (FITC-MPs, RITC-MPs) of the pristine P(S-co-MMA) and of the pristine dyes, FITC and RITC.

FTIR spectra of pristine dye-doped microparticles are shown in Figure 2. The spectra of the pristine polymer P(S-co-MMA) and pristine dyes (FITC and RITC) are also shown for comparison. In the spectrum of pristine P(S-co-MMA), peaks related to the styrene and MMA moieties are evidenced. In particular, peaks located between 3100 and 3000 cm^−1^ (aromatic C-H stretching), at 1951 and 1874 cm^−1^ (aromatic overtone bands), at 1606 cm^−1^ (aromatic C=C stretching), and at 756 and 701 cm^−1^ (aromatic out-of-plane C-H bending) are due to the benzene ring of styrene; peaks located between 3000 and 2800 cm^−1^ (alifatic C-H stretching), at 1737 cm^−1^ (C=O stretching), 1425 and 1386 cm^−1^ (alifatic C-H bending), and 1238 cm^−1^ (C-O stretching) are due to MMA [43]. The FTIR spectra of pristine FITC and RITC are rather complex, with many skeletal vibrations related to the polynuclear aromatic backbones of the two molecules. The spectrum of FITC also shows intense bands related to O-H stretching (3380 cm^−1^), C=O strecthing (1594 cm^−1^), and the vibrations of the NCS group (2034 cm^−1^). The spectrum of the FITC-doped microparticles (FITC-MPs) clearly shows these three bands superimposed on the spectrum of pristine P(S-co-MMA) as a clear indication of the successful immobilization of FITC in the microparticles, while the structure of the polymer remains unchanged. In the spectrum of the RITC-doped microparticles (RITC-MPs) the peaks related to pristine P(S-co-MMA) can also be clearly distinguished, indicating that the structure of the pristine polymers remains unaltered.

**Figure 3:**
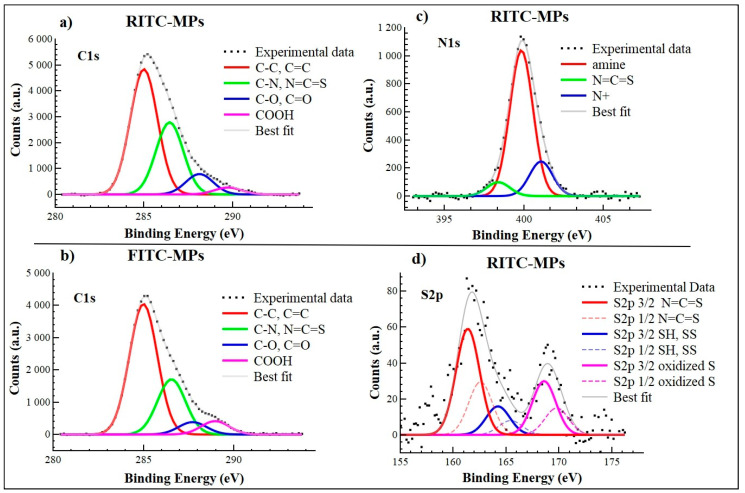
XPS spectra collected at C1s core level from (**a**) RITC-MPs and (**b**) FITC-MPs; (**c**) N1s and (**d**) S2p spectra collected from RITC-MPs.

X-ray photoelectron spectroscopy measurements allowed us to probe the presence of the dyes after the treatment. Spectra were collected at C1s, N1s, S2p, and O1s core levels (all BE (eV), FWHM (eV), atomic percentages, and proposed signal assignments are reported in SI Table S3). All the individuated spectral components confirmed RITC and FITC presence in MPs. In the C1s spectra of both samples (Figure 3a,b), the main component at 285.00 eV is associated with aliphatic and aromatic carbons; the second one, centered at 286.5 eV for RITC-MPs and 286.6 eV for FITC-MPs confirms the presence of C-N bonds and NCS functional groups that are characteristic of both dyes [46]. Components at higher BE are ascribed at C-O, C=O, and COOH in agreement with the chemical structure of dyes and microplastics. To obtain a better insight into the dyes’ molecular structure, spectra of N1s and S2p core levels of RITC-MPs and FITC-MPs were also analysed. N1s and S2p core-level spectra of RITC-MPs are shown in Figure 3c,d and are representative for both samples. The N1s spectrum of RITC-MPs (Figure 3c) has three components at 398.4 eV, 399.9 eV, and 401.1 eV BE associated with -N=C=S, NR_3_, and N^+^, respectively, in agreement with the RITC structure. Similarly, the N1s spectrum of FITC-MPs presents the component ascribed to -N=C=S functional groups at 398.8 eV BE. In the S2p spectra (Figure 3d), RITC-MPs are composite and very similar for both samples, having the spin-orbit pair relative to N=C=S group at low binding energy (S2p_3/2_ BE at about 161 eV), while signals at higher binding energy suggest the presence of disulphide S-S groups (S2p_3/2_ BE = 164 eV) and a partial oxidation of the dyes’ sulfur atoms (SOx species, S2p_3/2_ BE around 168 eV) [47,48].

### 3.3. Bioimaging in Test Organism

**Figure 4:**
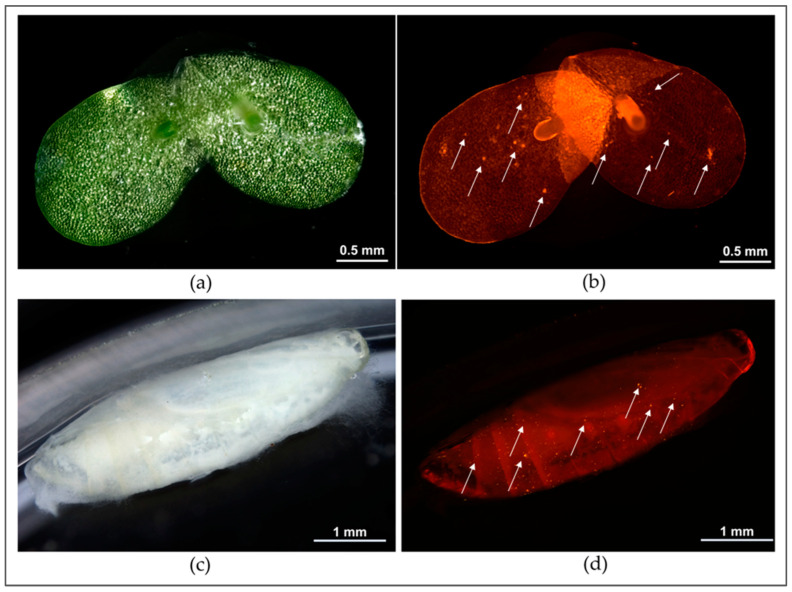
Light (**a**,**c**) and fluorescence (**b**,**d**) images of specimens of *L. minuta* (**a**,**b**) and *C. lemnata* (**c**,**d**); fluorescent microplastics are indicated with arrows.

The analysis of the fluorescence of specimens of *L. minuta* and larvae of *C. lemnata* exposed to RITC-MPs revealed the presence of some of these microparticles adsorbed on the body surfaces of both organisms (Figure 4b,d). The easy detectability of RITC-MPs on these organisms was evident in comparative observations of the same specimens observed by optical and fluorescence microscopy (Figure 4a–d).

The proposed method, easy and cheap, makes the microplastics fluorescent and therefore easily traceable, easily realizing bioimaging. In perspective, all this can considerably facilitate environmental studies of ecotoxicology and biomagnification focused on the analysis of the toxic effects of plastic contaminants in organisms. Indeed, with these RITC-MPs it is easy to localize them in an exposed organism and this can enable a better understanding of the adsorption, absorption, and bioaccumulation processes of the contaminants. Furthermore, the use of these fluorescent MPs makes it possible to identify the parts of the body (tissues/organs) of an organism that are most targeted by intoxication.

## 4. Conclusions

MPs available for laboratory studies to evaluate their environmental impact are scarce and expensive, especially if traceability via fluorescence is desired. In this work, a simple and cheap preparation was made to obtain microparticles of the commercial co-polymer P(S-co-MMA) doped with two different fluorescent dyes, RITC and FITC, widely used in the biological field for bioimaging. An osmosis-based method (OBM) was used and particles in the range of 0.1–9 microns were obtained, loaded with RITC and FITC (loading efficiency η (%) = 99% and 96% respectively). The MPs were characterized by SEM microscopy and FTIR and XPS spectroscopies. Furthermore, tests on aquatic organisms demonstrated the possible uses of these fluorescent MPs, in particular RITC-MPs, for bioimaging studies. In fact, thanks to their fluorescence, they were easily traced to verify their adsorption/absorption by model organisms, such as those used in this study.

## Figures and Tables

**Table 1:** Dimensions (expressed as Dw and Dn, the weight average diameter and the number average diameter, respectively) and polydispersity index (PI) of MPs and dye-doped MPs obtained using two different solvents (acetone, DMF) by OBM, using 7 mL of solution in dialysis membrane (* note: dye = 0.003 g).

Solvent	P(S-co-MMA) (g)	Dw (µm)	Dn (µm)	PI = Dw/Dn
Acetone (10 mL)	0.0530	1.95	1.98	0.98
Acetone (10 mL)	0.3015	2.32	2.36	0.98
DMF (10 mL)	0.0530	0.70	0.72	0.98
DMF (10 mL)	0.3015	0.93	0.93	1.00
Acetone (100 mL)	0.3015 + RITC *	2.04	2.08	0.98
Acetone (10 mL)	0.3015 + FITC *	9.17	9.19	0.99

## Data Availability

Not applicable.

## Supplementary Materials

The following supporting information can be downloaded at: https://www.mdpi.com/article/10.3390/polym15153245/s1, Table S1: Experimental conditions and SEM image of polymeric particles obtained by OBM, using polymer dissolved in solvent (acetone or DMF), put in dialysis membrane and immersed in 200 mL of distilled water for 24 h at room temperature; Table S2: Experimental conditions and SEM image of polymeric particles obtained by OBM, using different amounts of polymer dissolved with different amounts of dye (RITC or FITC) in different volumes of acetone, put in dialysis membrane and immersed in 200 mL of distilled water for 24 h at room temperature; Table S3: Spectra collected at C1s, N1s, S2p, and O1s core levels, all BE (eV), FWHM (eV), atomic percentages, and proposed signal assignments; Figure S1: Calibration curves for RITC and FITC.
