# Supplementary material for: Dye-Doped Polymeric Microplastics: Light Tools for Bioimaging in Test Organisms
Source: Polymers (Basel). 2023 Jul 30;15(15):3245. doi: 10.3390/polym15153245 (PMC10422618; doi:10.3390/polym15153245)

Supplementary Materials
Dye-Doped Polymeric Microplastics: Light Tools for Bioimaging in Test Organisms

Federica Bertelà [1], Chiara Battocchio [1], Giovanna Iucci [1], Simona Ceschin [1,2], Dario Di Lernia [1], Flaminia Mariani [1], Andrea Di Giulio [1,2], Maurizio Muzzi [1] and Iole Venditti [1,*]

[1] Department of Sciences, Roma Tre University, via della Vasca Navale 79, 00146 Rome, Italy; federica.bertela@uniroma3.it (F.B.); chiara.battocchio@uniroma3.it (C.B.); giovanna.iucci@uniroma3.it (G.I.); simona.ceschin@uniroma3.it (S.C.); dar.dilernia@stud.uniroma3.it (D.D.L.); flaminia.mariani@uniroma3.it (F.M.); andrea.digiulio@uniroma3.it (A.D.G.); maurizio.muzzi@uniroma3.it (M.M.); iole.venditti@uniroma3.i (I.V.)
[2] NBFC, National Biodiversity Future Center, 90133 Palermo, Italy
* Correspondence: iole.venditti@uniroma3.it

Table S1. Experimental conditions and SEM image of polymeric particles obtained by OBM, using polymer dissolved in solvent (acetone or DMF), put 7 mL in dialysis membrane and immersed in 200 mL of distilled water for 5 days, at room temperature (25°C).

P(S-co-MMA) (g)	acetone (10 mL)	DMF (10 mL)
0.0530	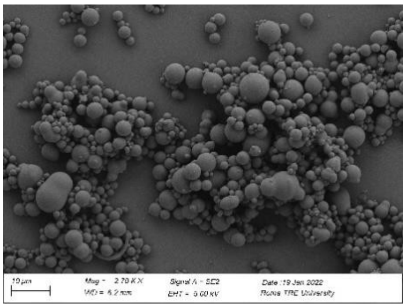	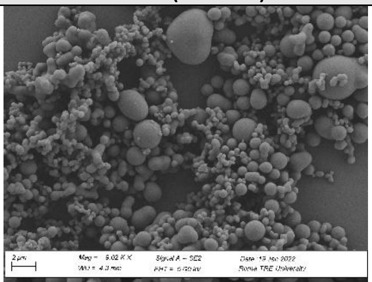 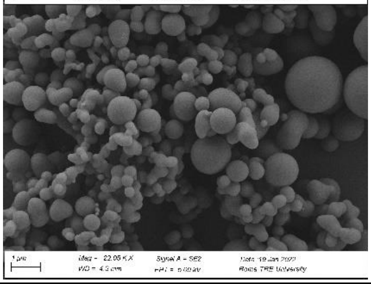
0.3015	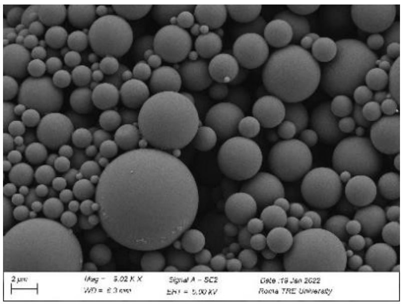	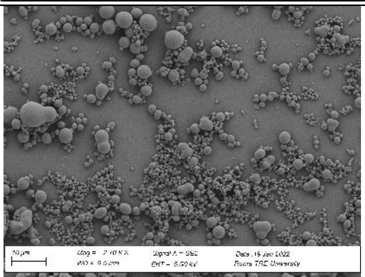 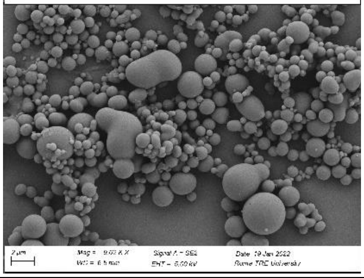

Table S2. Experimental conditions and SEM image of polymeric particles obtained by OBM, using different amount of polymer dissolved with different amount of dye (RITC or FITC) in different volume of acetone, put in dialysis membrane and immersed in 200 mL of distilled water for 5 days, at room temperature (25°C).

P(S-co-MMA) (g)	Acetone (mL)	RITC		FITC	
0.300	10	0.003 g	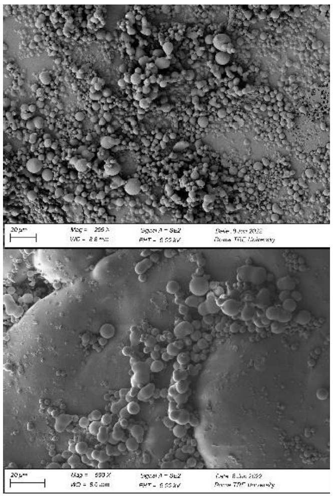	0.003mg	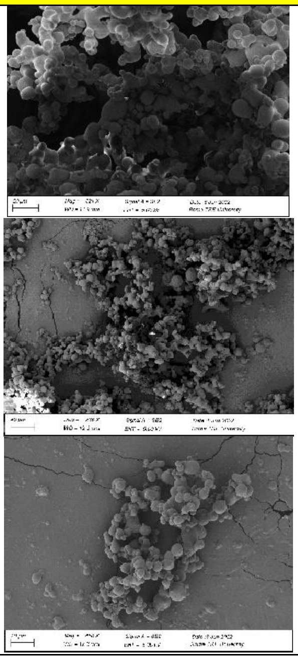
		0.030 g	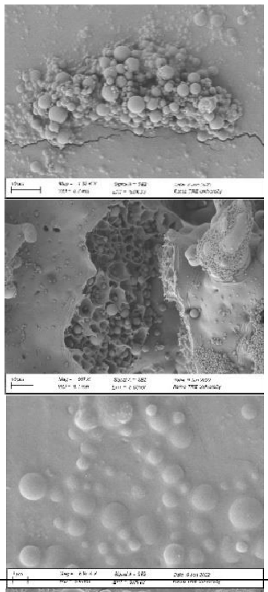	0.030 g	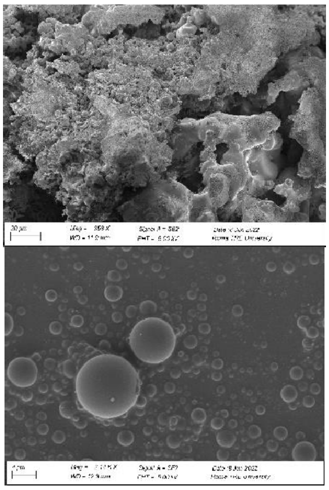
3.000 g	100	0.003mg	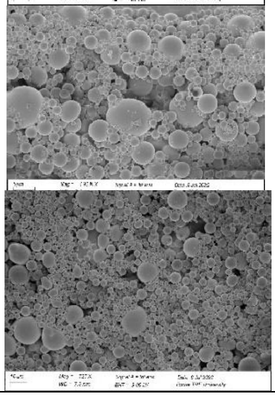	-	-

Table S3. Spectra were collected at C1s, N1s, S2p and O1s core levels (all BE (eV), FWHM (eV), atomic percentages and proposed signals assignments are here reported.

SAMPLE	SIGNAL	BE (eV)	FWHM (eV)	Atomic % (exp)	ASSIGNMENT
RITC + microplastics	C1s	285	1.8	37%	C-C, C=C
		286.5	1.8	13%	CN, NCS
		288.1	1.8	25%	C-O, C=O
		289.8	1.8	17%	COOH
	N1s	398.4	1.7	7%	NCS
		399.9	1.7	75%	amine
		401.1	1.7	18%	N^+
	S2p 3/2	161.4	2.6	56%	NCS
		164.2	2.6	15%	SS, SH
		168.6	2.6	29%	oxidized S
FITC + microplastics	C1s	285.0	1.8	38%	C-C
		286.6	1.8	13%	CN, NCS
		287.7	1.8	26%	C-O, C=O
		289.0	1.8	14%	COOH
	N1s	398.8	1.8	7%	NCS
		400.3	1.8	74%	amine
		401.6	1.8	19%	N^+
	$S2p_{3/2}$	160.8	3.0	13%	NCS
		164.0	3.0	57%	SH, S-S
		168.9	3.0	22%	oxidized S
		172.2	3.0	8%	oxidized S

Figure S1. Calibration curves for RITC (a) and FITC (b).

(a)

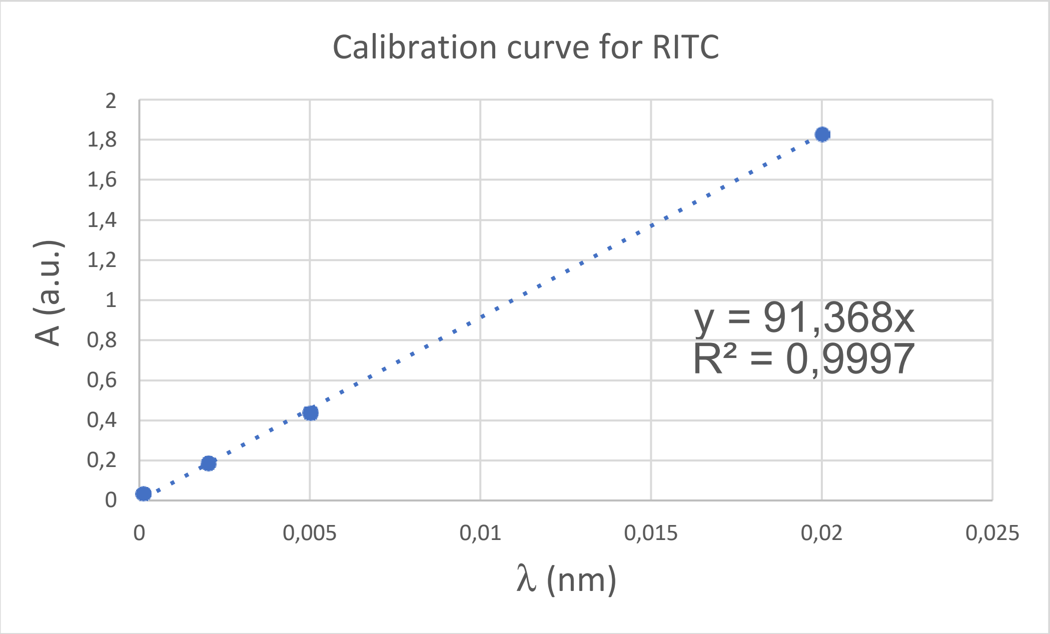

(b)

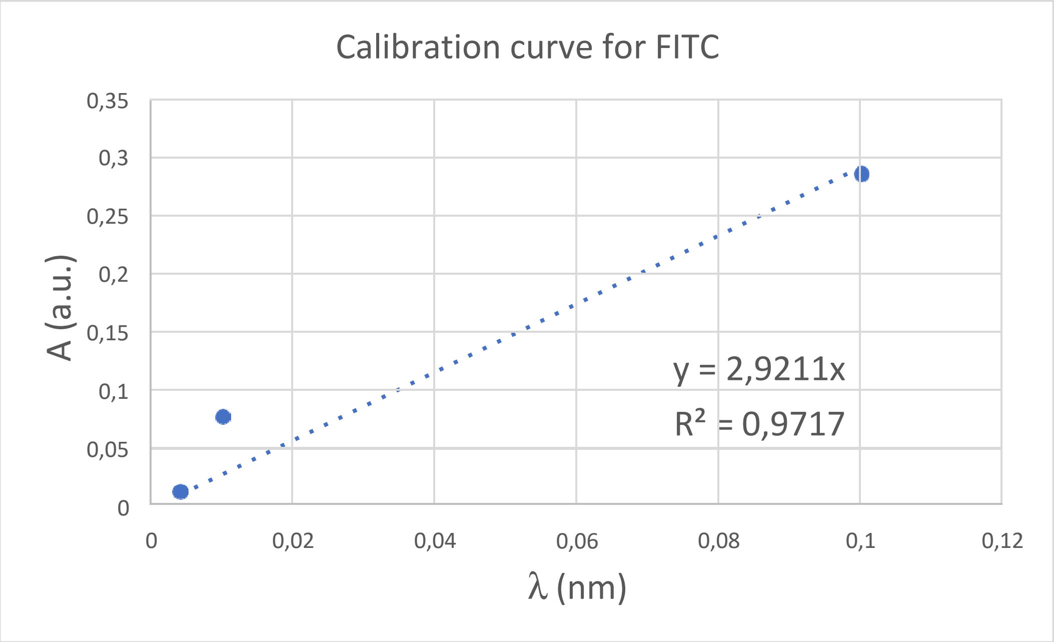

Supplement: Supplementary file 1 [file polymers-15-03245-s001.zip › polymers-2494055-supplementary.pdf]
